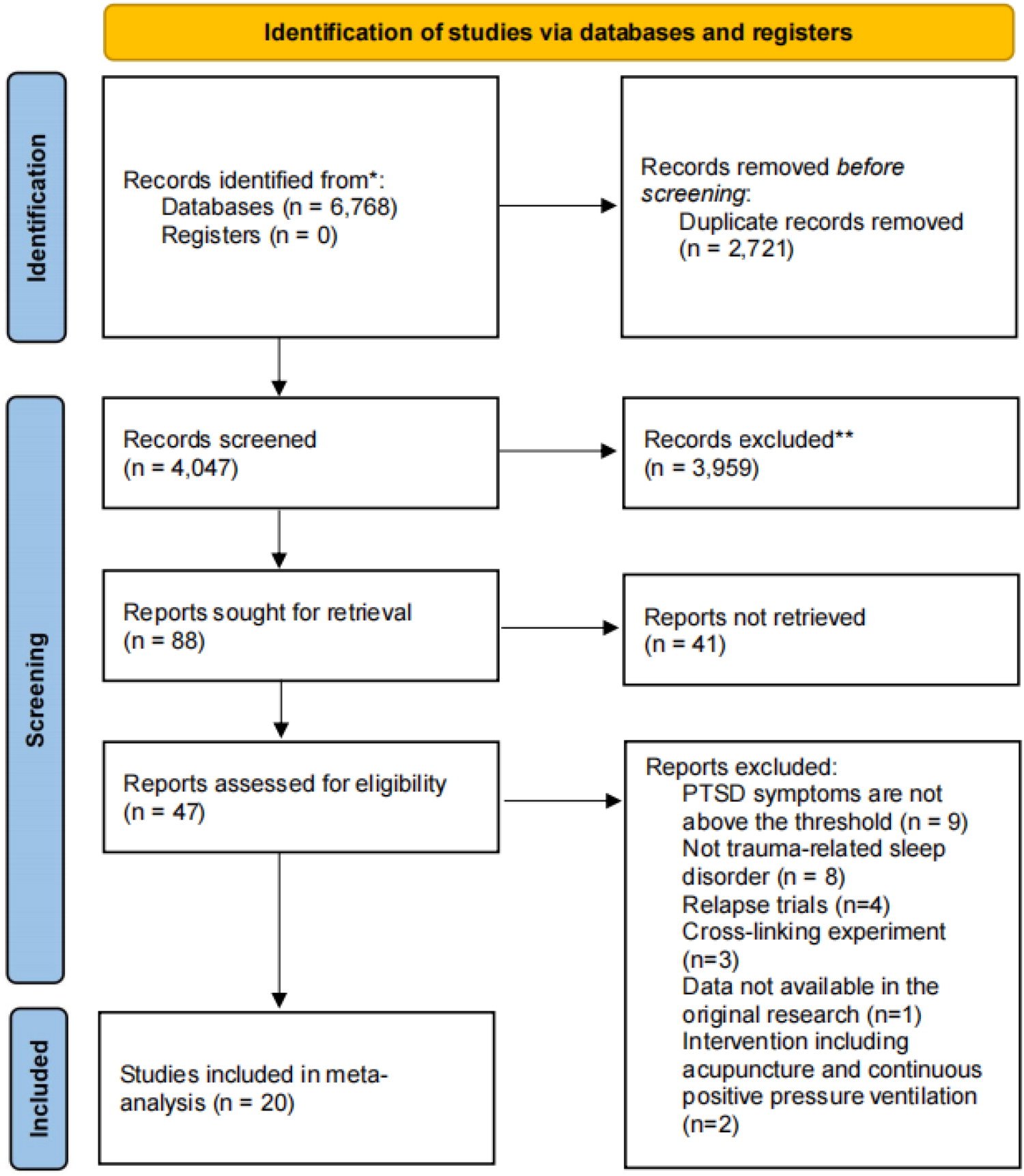# Correction

**DOI:** 10.1080/07853890.2026.2722864

**Published:** 2026-08-26

**Article title:** Psychotherapeutic and pharmacological agents for post-traumatic stress disorder with sleep disorder: network meta-analysis

**Authors:** Huang, C. Y., Zhao, Y. F., Zhang, Z. X., Liu, R. B., Liu, J. L., Li, X. Z., Luo, J., Yue, L., Zhang, C.

**Journal:** *Annals of Medicine*

**Bibliometrics:** Volume 56, Number 01, 2381696

**DOI:** https://doi.org/10.1080/07853890.2024.2381696

During preparation of the manuscript for submission, the search retrieval results were updated. The manuscript files were updated to reflect the final search results as detailed in Supplementary Method 1, but an oversight led to updates being inadvertently missed in a section of the main text and a figure. The rest of the subsequent results were correct, and other results were not affected.

**Text correction**

The first paragraph of Section 3.1 was previously published with uncorrected search result numbers. The numbers have now been updated.

Previously published as:

The search yield 5,095 results. After the initial evaluation, 1,048 duplicates were removed and 4,026 studies were regarded as irrelevant (as determined by reading the titles and abstracts). 21 articles with 24 RCTs were determined by reading the full text. Ultimately, 20 studies were included in the NMA. Supplementary Figure 1 shows the work flow for the selection of studies.

Corrected to:

The search yield 6,768 results. After the initial evaluation, 2,721 duplicates were removed, and 4,026 studies were regarded as irrelevant (as determined by reading the titles and abstracts). 20 articles with 24 RCTs were determined by reading the full text. Ultimately, 20 studies were included in this NMA. **Supplementary Figure 1** shows the workflow for the selection of studies.

**Figure correction**

Supplementary Figure 1 was previously published with uncorrected search result numbers. The numbers have now been updated.

Previously published as:


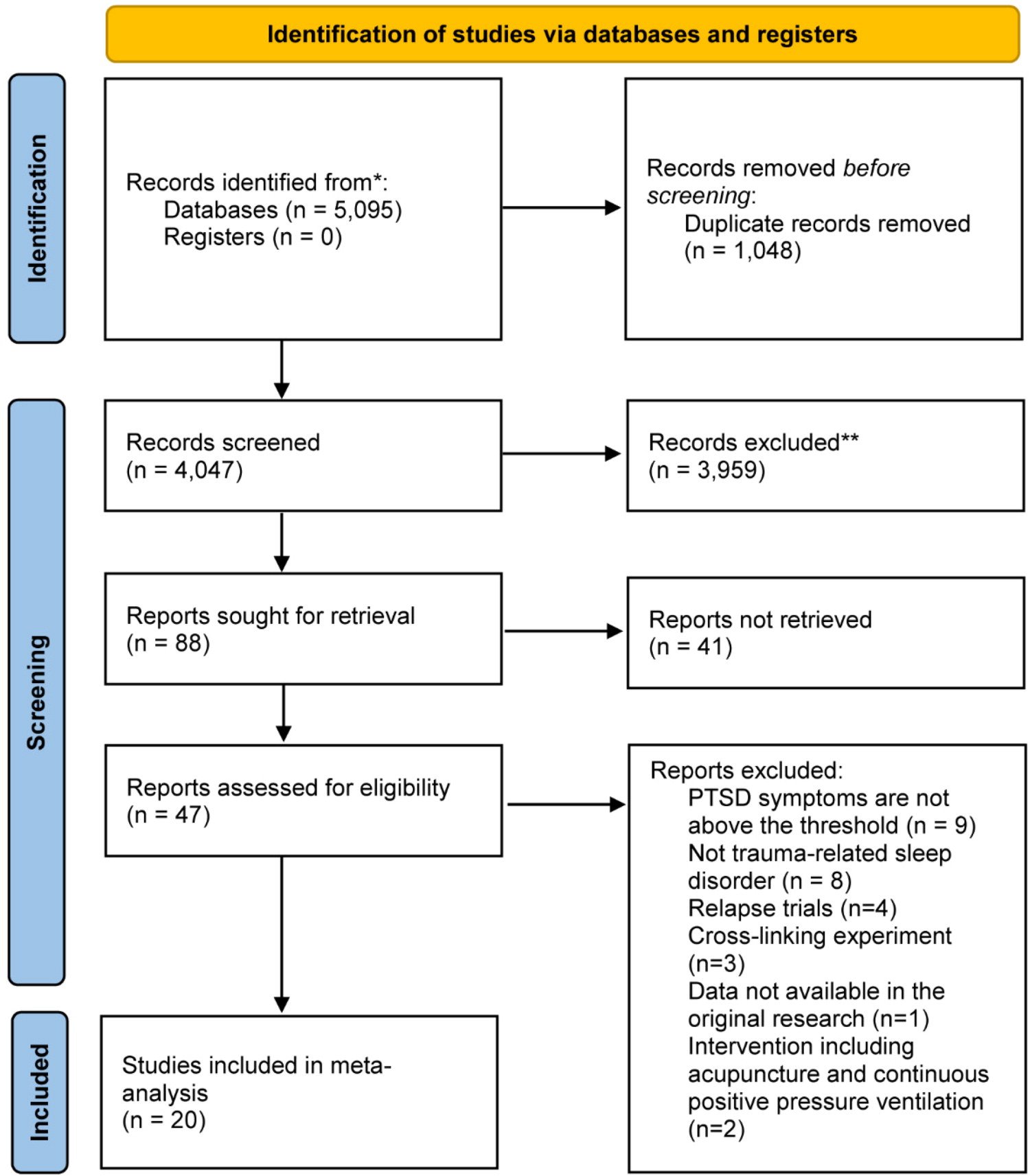


Corrected to: